# Supplementary material for: Regression with race-modifiers: towards equity and interpretability
Source: medRxiv. 2024 Feb 28:2024.01.04.23300033. Preprint. [Version 3] doi: 10.1101/2024.01.04.23300033 (PMC10925363; doi:10.1101/2024.01.04.23300033)
Supplement: Supplementary file 1 [file NIHPP2024.01.04.23300033v3-supplement-1.pdf]

medRxiv preprint doi: https://doi.org/10.1101/2024.01.04.23300033; this version posted February 28, 2024. The copyright holder for this preprint
(which was not certified by peer review) is the author/funder, who has granted medRxiv a license to display the preprint in perpetuity.
It is made available under a CC-BY 4.0 International license .

A. Supporting Information

To prove Theorems 1 and 2, we leverage a classical property of OLS.

Frisch-Waugh-Lovell (FWL) Theorem. For a partition of the $n \times p$ covariate matrix $\boldsymbol{X} = (\boldsymbol{X}_0 : \boldsymbol{X}_1)$ into p_0 and p_1 columns, the partition of the ordinary least squares estimator $\hat{\boldsymbol{\beta}} = (\hat{\boldsymbol{\beta}}_0^\top, \hat{\boldsymbol{\beta}}_1^\top)^\top$ satisfies $\hat{\boldsymbol{\beta}}_0 = (\boldsymbol{X}_0^\top \boldsymbol{E}_{01})^{-1}\boldsymbol{E}_{01}^\top \boldsymbol{y} = (\boldsymbol{E}_{01}^\top \boldsymbol{E}_{01})^{-1}\boldsymbol{E}_{01}^\top \boldsymbol{y}$, where $\boldsymbol{E}_{01} = (\boldsymbol{I}_n - \boldsymbol{H}_{X_1})\boldsymbol{X}_0$ is the $n \times p_0$ matrix of residuals from regressing each column of $\boldsymbol{X}_0$ on $\boldsymbol{X}_1$, $\boldsymbol{H}_{X_1} = \boldsymbol{X}_1(\boldsymbol{X}_1^\top \boldsymbol{X}_1)^{-1}\boldsymbol{X}_1^\top$ is the corresponding hat matrix for $\boldsymbol{X}_1$, and $\boldsymbol{y} = (y_1, \ldots, y_n)^\top$ is the vector of outcomes.

Although Theorems 1 and 2 and stated in terms of approximate equivalence under approximate equal-variance conditions, here we prove exact equivalence under exact equal-variance conditions.

Proof (Theorem 1). Let $\boldsymbol{y} = (y_1, \ldots, y_n)^\top$, $\boldsymbol{x} = (x_1, \ldots, x_n)^\top$, and $\boldsymbol{Z}$ be the matrix of categorical dummy variables with entries $[\boldsymbol{Z}]_{ir} = 1$ if $r_i = r$ and zero otherwise. Using the FWL Theorem, the estimated coefficients under the main-only model satisfy $\hat{\alpha}_1^M = (\boldsymbol{x}^\top \hat{\boldsymbol{e}}_{x\sim r})^{-1}\hat{\boldsymbol{e}}_{x\sim r}^\top \boldsymbol{y}$, where $\hat{\boldsymbol{e}}_{x\sim r}$ is the vector of residuals from regressing the continuous variable $\{x\}_{i=1}^n$ on the categorical variable $\{r_i\}_{i=1}^n$ (i.e., $\boldsymbol{Z}$). Similarly, the estimated coefficients under the race-modified model satisfy $\hat{\alpha}_1 = (\boldsymbol{x}^\top \hat{\boldsymbol{e}}_{x\sim r+Z_{XQ}})^{-1}\hat{\boldsymbol{e}}_{x\sim r+Z_{XQ}}^\top \boldsymbol{y}$, where $\hat{\boldsymbol{e}}_{x\sim r+Z_{XQ}}$ are the residuals from regressing the continuous variable $\{x\}_{i=1}^n$ on the categorical variable $\{r_i\}_{i=1}^n$ (i.e., $\boldsymbol{Z}$) *and* the reparametrized interaction term that enforces ABCs, $\boldsymbol{Z}_{XQ} = \boldsymbol{Z}_X \boldsymbol{Q}_{-(1:m)}$, where $\boldsymbol{Z}_X = \boldsymbol{D}_X \boldsymbol{Z}$ and $\boldsymbol{D}_X = \mathrm{diag}(\boldsymbol{x})$. Thus, it suffices to show that $\hat{\boldsymbol{e}}_{x\sim r} = \hat{\boldsymbol{e}}_{x\sim r+Z_{XQ}}$, which occurs when the additional (interaction) coefficients from the latter model, say $\hat{\boldsymbol{b}}_{Z_{XQ}}$ (corresponding to $\boldsymbol{Z}_{XQ}$), are identically zero. Again using the FWL Theorem, these estimated coefficients are $\hat{\boldsymbol{b}}_{Z_{XQ}} = \boldsymbol{Q}_{-(1:m)}(\boldsymbol{Z}_{XQ}^\top \boldsymbol{E}_{Z_{XQ}})^{-1}\boldsymbol{E}_{Z_{XQ}}^\top \boldsymbol{x}$, where $\boldsymbol{E}_{Z_{XQ}}$ is the matrix of residuals from regressing $\boldsymbol{Z}_{XQ}$ on $\boldsymbol{Z}$, i.e., $\boldsymbol{E}_{Z_{XQ}} = \boldsymbol{Z}_{XQ} - \boldsymbol{H}_Z \boldsymbol{Z}_{XQ}$ where $\boldsymbol{H}_Z = \boldsymbol{Z}(\boldsymbol{Z}^\top \boldsymbol{Z})^{-1}\boldsymbol{Z}^\top$ is the hat matrix for the categorical covariate. Thus, showing $\boldsymbol{x}^\top \boldsymbol{E}_{Z_{XQ}} = \boldsymbol{0}$ is sufficient. Factoring $\boldsymbol{x}^\top \boldsymbol{E}_{Z_{XQ}} = (\boldsymbol{x}^\top \boldsymbol{Z}_X - \boldsymbol{x}^\top \boldsymbol{H}_Z \boldsymbol{Z}_X)\boldsymbol{Q}_{-(1:m)}$, first observe that $\boldsymbol{x}^\top \boldsymbol{Z}_X = \boldsymbol{x}^\top \boldsymbol{D}_X \boldsymbol{Z} = (\ldots, s_r^2, \ldots)$ is the vector of $s_r^2 = \sum_{r_i = r} x_i^2$ across groups. Next, observe that $(\boldsymbol{Z}^\top \boldsymbol{Z})^{-1}\boldsymbol{Z}^\top \boldsymbol{Z}_X = \mathrm{diag}(\{\bar{x}_r\}_r)$ contains the sample means of $\{x\}_{i=1}^n$ by each group r, and therefore $\boldsymbol{x}^\top \boldsymbol{H}_Z \boldsymbol{Z}_X = \boldsymbol{x}^\top \boldsymbol{Z}\mathrm{diag}(\{\bar{x}_r\}_r) = (\ldots, n_r \bar{x}_r^2, \ldots)$ with $n_r = n\hat{\pi}_r$. Combining these results, we have $\boldsymbol{x}^\top \boldsymbol{E}_{Z_{XQ}} = \boldsymbol{v}^\top \boldsymbol{Q}_{-(1:m)}$, where $\boldsymbol{v}^\top = (\ldots, s_r^2 - n_r \bar{x}_r^2, \ldots) = n(\ldots, \hat{\pi}_r \hat{\sigma}_{x[r]}^2, \ldots) = n\hat{\sigma}_{x[1]}^2 \hat{\boldsymbol{\pi}}^\top$ under the assumption that $\hat{\sigma}_{x[r]}^2 = \hat{\sigma}_{x[1]}^2$ is common for all r. Finally, the definition of $\boldsymbol{Q}_{-(1:m)}$ via ABCs implies that $\hat{\boldsymbol{\pi}}^\top \boldsymbol{Q}_{-(1:m)} = \boldsymbol{0}$, which proves the result. □

Proof (Theorem 2). Let $\boldsymbol{X}$ denote the $n \times p$ matrix of continuous covariates, $\boldsymbol{Z}$ the matrix of categorical dummy variables with entries $[\boldsymbol{Z}]_{ir} = 1$ if $r_i = r$ and zero otherwise, and $\boldsymbol{Z}_{XQ} = (\boldsymbol{Z}_{X_1 Q}, \ldots, \boldsymbol{Z}_{X_p Q})$ with $\boldsymbol{Z}_{X_j Q} = \boldsymbol{Z}_{X_j}\boldsymbol{Q}_{-(1:m)}$, $\boldsymbol{Z}_{X_j} = \boldsymbol{D}_{X_j}\boldsymbol{Z}$, and $\boldsymbol{D}_{X_j} = \mathrm{diag}(\boldsymbol{x}_j)$. By the FWL Theorem, it suffices to show that $\boldsymbol{E}_M = \boldsymbol{E}$, where $\boldsymbol{E}_M = (\boldsymbol{I}_n - \boldsymbol{H}_Z)\boldsymbol{X}$ are the residuals from regressing each column of $\boldsymbol{X}$ on $\boldsymbol{Z}$ and $\boldsymbol{E}$ are similarly the residuals from regressing each column of $\boldsymbol{X}$ on $\boldsymbol{Z}$ *and* $\boldsymbol{Z}_{XQ}$. Thus, it is sufficient to show that the coefficients associated with $\boldsymbol{Z}_{XQ}$ in the latter regression are identically zero. Again using the FWL Theorem, we see that this occurs whenever $\boldsymbol{X}^\top \boldsymbol{E}_{Z_{XQ}} = \boldsymbol{0}$, where $\boldsymbol{E}_{Z_{XQ}} = \boldsymbol{Z}_{XQ} - \boldsymbol{H}_Z \boldsymbol{Z}_{XQ} = (\boldsymbol{Z}_{X_1 Q} - \boldsymbol{H}_Z \boldsymbol{Z}_{X_1 Q}, \ldots, \boldsymbol{Z}_{X_p Q} - \boldsymbol{H}_Z \boldsymbol{Z}_{X_p Q})$. Noticing that $\boldsymbol{X}^\top \boldsymbol{E}_{Z_{XQ}} = (\boldsymbol{X}^\top(\boldsymbol{Z}_{X_1 Q} - \boldsymbol{H}_Z \boldsymbol{Z}_{X_1 Q}), \ldots, \boldsymbol{X}^\top(\boldsymbol{Z}_{X_p Q} - \boldsymbol{H}_Z \boldsymbol{Z}_{X_p Q}))$, we consider the individual components $\boldsymbol{x}_h^\top(\boldsymbol{Z}_{X_j Q} - \boldsymbol{H}_Z \boldsymbol{Z}_{X_j Q}) = (\boldsymbol{x}_h^\top \boldsymbol{D}_{x_j}\boldsymbol{Z} - \boldsymbol{x}_h^\top \boldsymbol{H}_Z \boldsymbol{D}_{x_j}\boldsymbol{Z})\boldsymbol{Q}_{-(1:m)}$, each of which must equal the zero vector with dimension equal to

medRxiv preprint doi: https://doi.org/10.1101/2024.01.04.23300033; this version posted February 28, 2024. The copyright holder for this preprint (which was not certified by peer review) is the author/funder, who has granted medRxiv a license to display the preprint in perpetuity.
It is made available under a CC-BY 4.0 International license .

the number of categories. Noting that $\boldsymbol{x}_h^\top \boldsymbol{D}_{x_j} \boldsymbol{Z} = (\ldots, s_r(j,h), \ldots)$ with $s_r(j,h) = \sum_{r_i=r} x_{ij} x_{ih}$ and $\boldsymbol{x}_h^\top \boldsymbol{H}_Z \boldsymbol{D}_{x_j} \boldsymbol{Z} = (\ldots, n_r \bar{x}_r(j) \bar{x}_r(h), \ldots)$ with $\bar{x}_r(j) = n_r^{-1} \sum_{r_i=r} x_{ij}$, we apply the same arguments as in Theorem 1. □

Table A.1. Characteristics of the North Carolina data ($n = 27,638$).

Variable	Proportion
Mother's race	
NH White	57.9%
NH Black	36.1%
Hispanic	6.0%
Sex	
Male	50.0%
Female	50.0%
Mother's education level	
Did not complete high school	24.2%
Completed high school	36.8%
At least some postsecondary	39.0%
Mother's marital status	
Married at time of birth	56.2%
Not married at time of birth	43.8%
Mother's smoking status	
Smoker	16.9%
Non-smoker	83.1%
Economically disadvantaged	
Yes	61.0%
No	39.0%

Sample proportions by group for each categorical variable. These sample proportions are used for ABCs with each categorical variable. "Economically disadvantaged" is determined by participation in the National Lunch Program.

DRAFT

Table A.2. The (scaled) sample standard deviations $\hat{\sigma}_{x[r]}(j)$ by race r for each covariate $j = 1, \ldots, p$.

Variable j	$\hat{\sigma}_{x[\texttt{NHW}]}(j)$	$\hat{\sigma}_{x[\texttt{NHB}]}(j)$	$\hat{\sigma}_{x[\texttt{Hisp}]}(j)$
Blood lead level	0.949	1.043	0.976
$\text{PM}_{2.5}$ exposure	0.999	1.004	0.924
Racial isolation (RI)	0.688	1.063	0.936
Mother's age	0.998	0.974	0.886
Birthweight percentile for gestational age	0.998	0.955	0.984

The invariance result for estimators with and without race-modifiers (Theorem 2) requires $\hat{\sigma}_{x[\texttt{NHW}]}(j) = \hat{\sigma}_{x[\texttt{NHB}]}(j) = \hat{\sigma}_{x[\texttt{Hisp}]}(j)$ for each covariate j (and similarly for the cross-covariances). Although this condition is clearly violated, the estimates and SEs maintain invariance (Figure 2), which suggests strong empirical robustness for the desirable invariance property of ABCs.

medRxiv preprint doi: https://doi.org/10.1101/2024.01.04.23300033; this version posted February 28, 2024. The copyright holder for this preprint (which was not certified by peer review) is the author/funder, who has granted medRxiv a license to display the preprint in perpetuity.
It is made available under a CC-BY 4.0 International license .

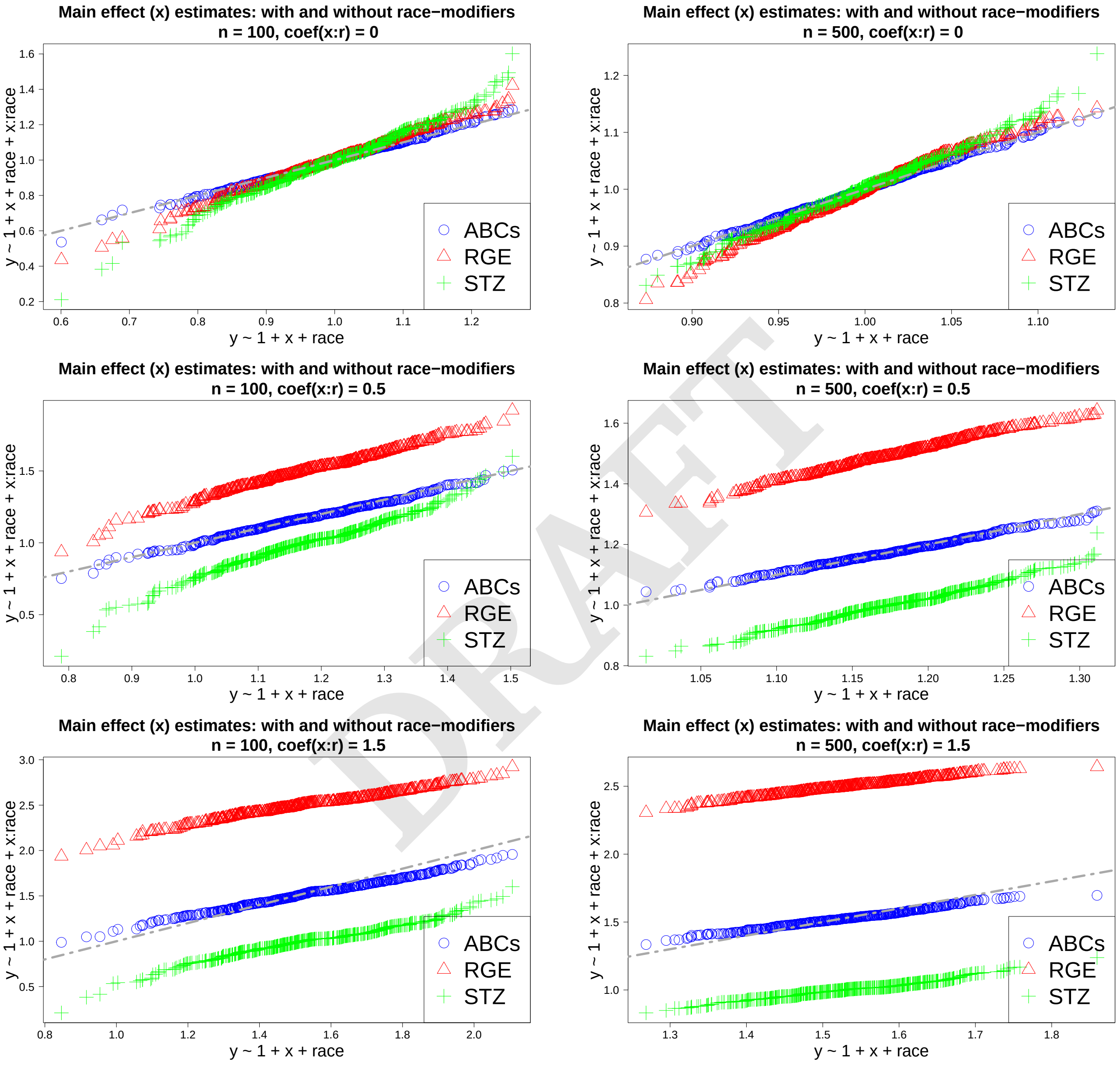

Fig. A.1. Estimated x-effects $(\hat{\alpha}_1^M, \hat{\alpha}_1)$ under different categorical encodings across 500 simulated datasets for $n = 100$ (left) and $n = 500$ (right) and varying race-modifier effects $\gamma \in \{0, 0.5, 1.5\}$ (top to bottom). Uniquely, ABCs produce nearly identical x-effect estimates with and without the race-modifier (45° line), which preserves the interpretations from the simpler (main-only) model.

medRxiv preprint doi: https://doi.org/10.1101/2024.01.04.23300033; this version posted February 28, 2024. The copyright holder for this preprint (which was not certified by peer review) is the author/funder, who has granted medRxiv a license to display the preprint in perpetuity.
It is made available under a CC-BY 4.0 International license .

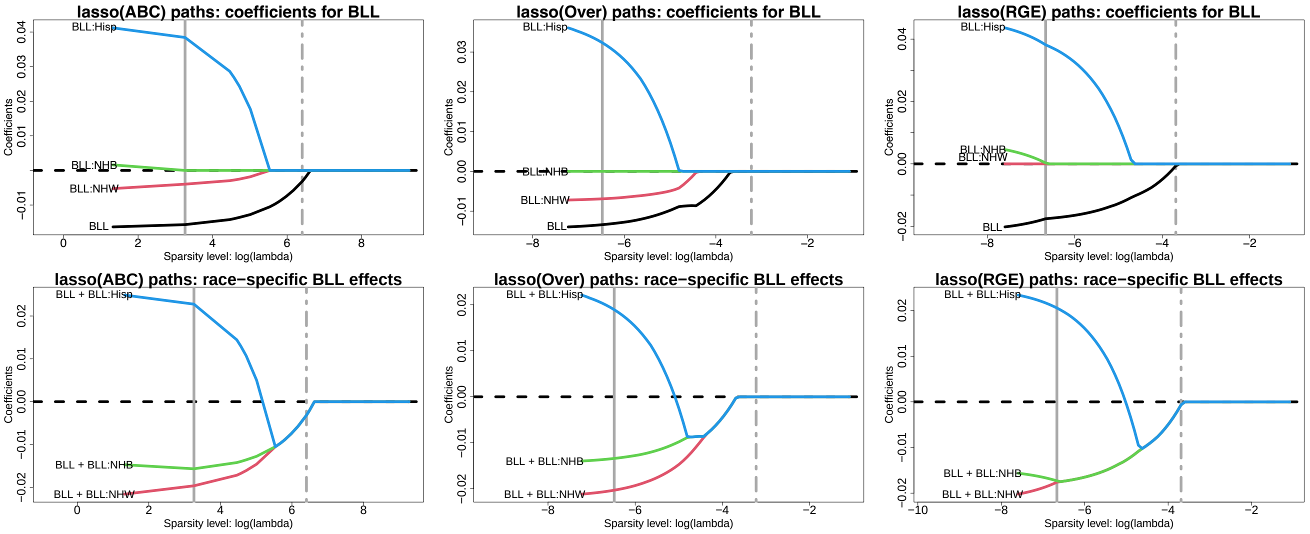

Fig. A.2. Estimated lasso paths for blood lead level (BLL) across varying sparsity levels ($\log \lambda$) for the model coefficients $\hat{\alpha}_{\texttt{BLL}}, \hat{\gamma}_{\texttt{BLL:r}}$ (top) and the race-specific slopes $\hat{\mu}'_{\texttt{BLL}}(r) = \hat{\alpha}_{\texttt{BLL}} + \hat{\gamma}_{\texttt{BLL:r}}$ (bottom) under ABCs (left), overparametrized estimation (center), and RGE (right); vertical lines identify λ for the minimum CV error (solid) and one-standard-error rule (dot-dashed). The outcome is 4th end-of-grade reading score and the covariates include all variables in Table 2. Small λ approximately corresponds to OLS, while increasing λ yields sparsity. Under RGE, the race-specific effects are pulled toward the NH White estimate (bottom right). For overparametrized estimation, the paths are similar to the ABC versions (center and left), but estimate $\hat{\gamma}_{\texttt{BLL:NHB}} = 0$ for all λ and thus implicitly selects NH Black as the reference group. This explains the differences from RGE, which uses a NH White reference group ($\hat{\gamma}_{\texttt{BLL:NHW}} = 0$). Under ABCs, the race-specific effects are pulled toward a global BLL effect (bottom left), which is is nonzero and detrimental for 4th end-of-grade reading scores.

DRAFT

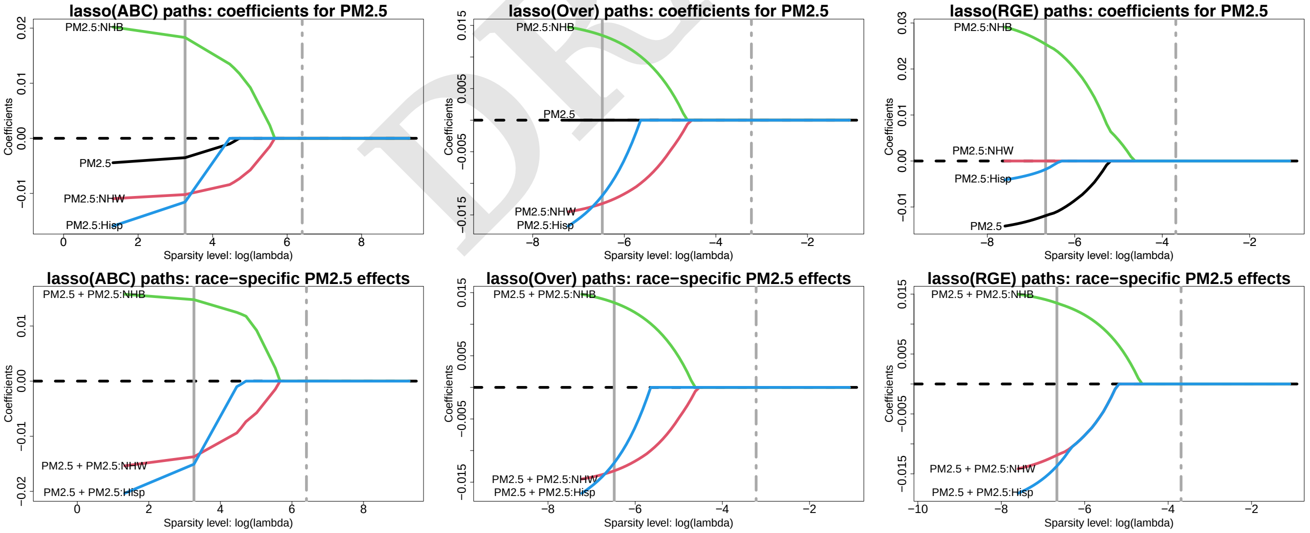

Fig. A.3. Estimated lasso paths for $PM_{2.5}$ exposure ($PM_{2.5}$) across varying sparsity levels ($\log \lambda$) for the model coefficients $\hat{\alpha}_{\texttt{PM2.5}}, \hat{\gamma}_{\texttt{PM2.5:r}}$ (top) and the race-specific slopes $\hat{\mu}'_{\texttt{PM}}(r) = \hat{\alpha}_{\texttt{PM2.5}} + \hat{\gamma}_{\texttt{PM2.5:r}}$ (bottom) under ABCs (left) overparametrized estimation (center), and RGE (right); vertical lines identify λ for the minimum CV error (solid) and one-standard-error rule (dot-dashed). The outcome is 4th end-of-grade reading score and the covariates include all variables in Table 2. Small λ approximately corresponds to OLS, while increasing λ yields sparsity. The ABC paths confirm the OLS output: the global $PM_{2.5}$ effect is pulled toward zero in advance of the race-specific deviations (top left), so the race-specific slopes merge at a global estimate of zero (bottom left). The RGE estimates demonstrate the shrinkage of race-specific effects toward the NH White estimate. The overparameterized paths for the race-specific effects resemble those for ABCs (bottom center and bottom left), but the overparametrized version sets the main effect to zero, $\hat{\alpha}_{\texttt{PM2.5}} = 0$ and thus results in different coefficients compared to either ABCs or RGE (top).

medRxiv preprint doi: https://doi.org/10.1101/2024.01.04.23300033; this version posted February 28, 2024. The copyright holder for this preprint (which was not certified by peer review) is the author/funder, who has granted medRxiv a license to display the preprint in perpetuity.
It is made available under a CC-BY 4.0 International license .

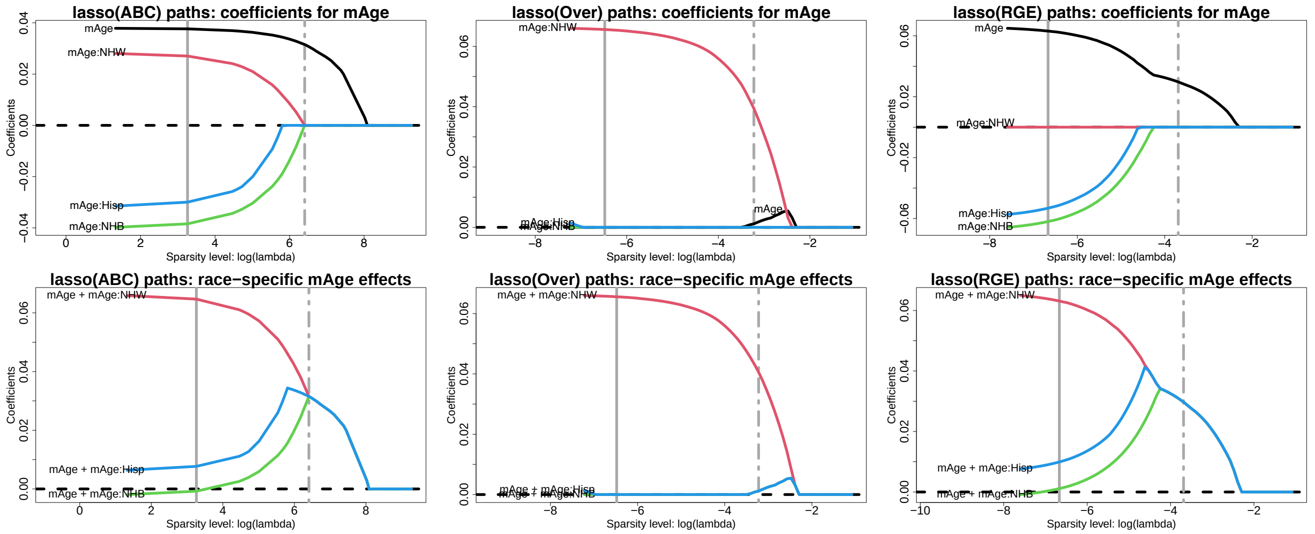

Fig. A.4. Estimated lasso paths for mother's age (mAge) across varying sparsity levels ($\log\lambda$) for the model coefficients $\hat{\alpha}_{\texttt{mAge}}, \hat{\gamma}_{\texttt{mAge:r}}$ (top) and the race-specific slopes $\hat{\mu}'_{\texttt{PM}}(r) = \hat{\alpha}_{\texttt{mAge}} + \hat{\gamma}_{\texttt{mAge:r}}$ (bottom) under ABCs (left) overparametrized estimation (center), and RGE (right); vertical lines identify λ for the minimum CV error (solid) and one-standard-error rule (dot-dashed). The outcome is 4th end-of-grade reading score and the covariates include all variables in Table 2. Small λ approximately corresponds to OLS, while increasing λ yields sparsity. The racial bias of RGE is clear (bottom right): the race-specific effects are each pulled toward the NH White estimate. By comparison, under ABCs, the race-specific effects are pulled toward a global mAge effect (bottom left), which is nonzero and positive for 4th end-of-grade reading scores. The overparametrized estimation cannot determine a reference group, and exhibits erratic behavior that does not resemble either alternative.

DRAFT

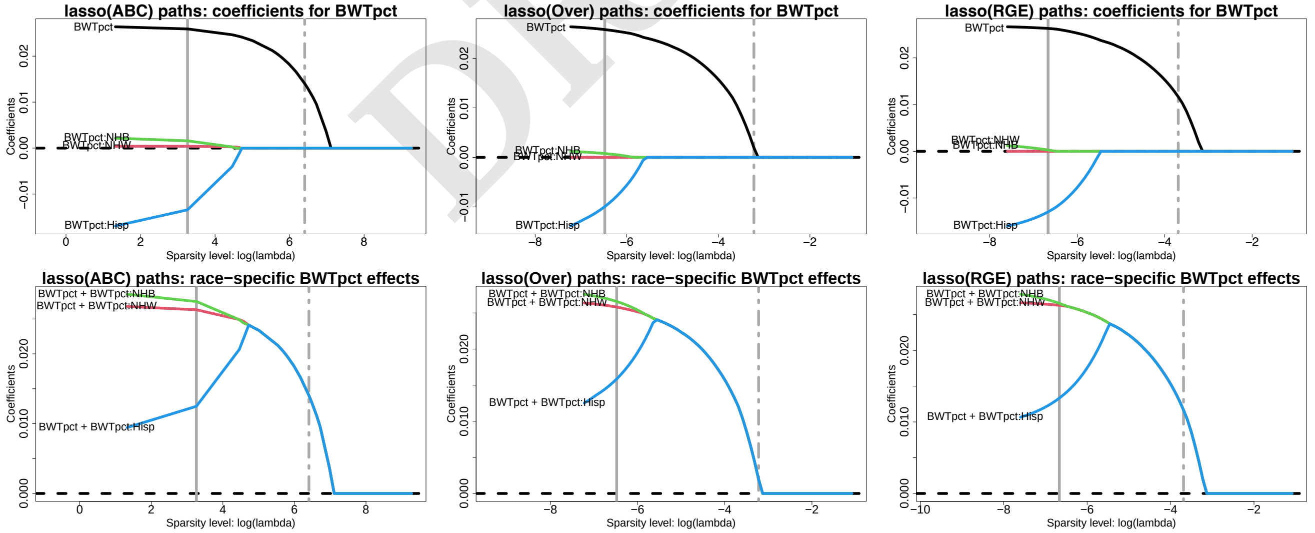

Fig. A.5. Estimated lasso paths for birthweight percentile for gestational age (BWTpct) across varying sparsity levels ($\log\lambda$) for the model coefficients $\hat{\alpha}_{\texttt{BWTpct}}, \hat{\gamma}_{\texttt{BWTpct:r}}$ (top) and the race-specific slopes $\hat{\mu}'_{\texttt{PM}}(r) = \hat{\alpha}_{\texttt{BWTpct}} + \hat{\gamma}_{\texttt{BWTpct:r}}$ (bottom) under ABCs (left) overparametrized estimation (center), and RGE (right); vertical lines identify λ for the minimum CV error (solid) and one-standard-error rule (dot-dashed). The outcome is 4th end-of-grade reading score and the covariates include all variables in Table 2. Small λ approximately corresponds to OLS, while increasing λ yields sparsity. Under ABCs, the race-specific effects are all positive, and merge at a positive global effect of BWTpct. Because the race-specific deviations for NH White individuals are near zero (top left), the RGE paths—which fix these coefficients at zero by design—are very similar to the ABC paths. This effect is similar for overparametrized estimation.

medRxiv preprint doi: https://doi.org/10.1101/2024.01.04.23300033; this version posted February 28, 2024. The copyright holder for this preprint (which was not certified by peer review) is the author/funder, who has granted medRxiv a license to display the preprint in perpetuity.
It is made available under a CC-BY 4.0 International license .

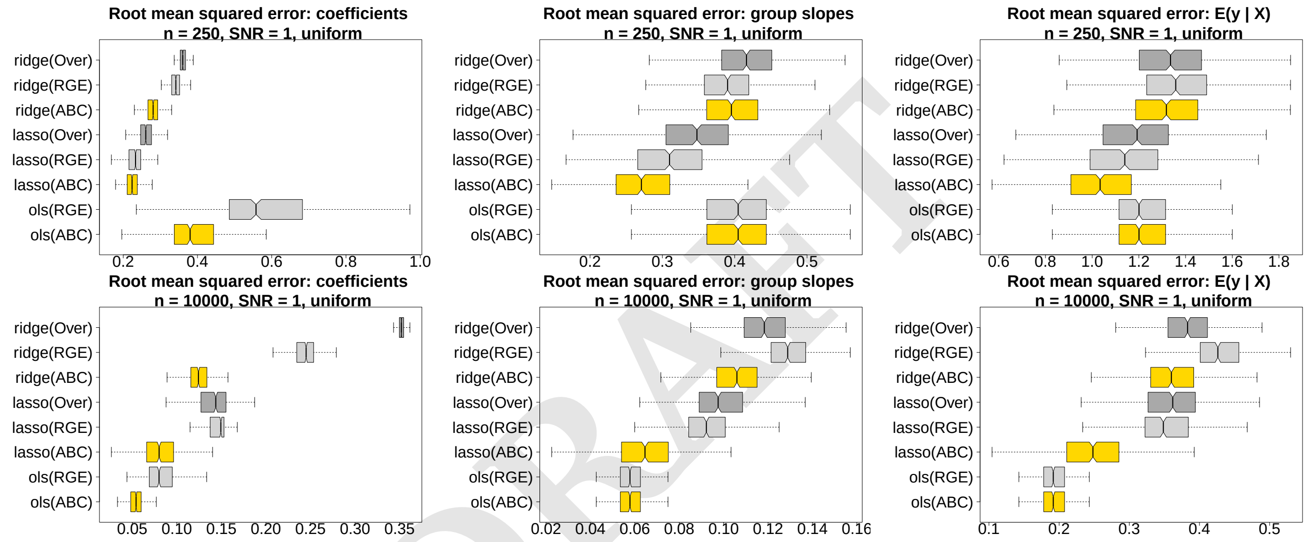

Fig. A.6. Estimation and prediction accuracy for the regression coefficients (left), the race-specific slopes (center), and the fitted values (right) for $n = 250$ (top) and $n = 10{,}000$ (bottom) across 500 simulated datasets; nonoverlapping notches indicate significant differences between medians. Data are generated from a Gaussian main-only model with $p = 10$ covariates and a categorical variable with uniform proportions $\boldsymbol{\pi} = (0.25, 0.25, 0.25, 0.25)^\top$; both RGE and ABCs are satisfied in the true data-generating process. All fitted models use the race-modified model Eq. (1). ABCs (gold) outperform both RGE (light gray) and `Over` (dark gray) within each estimation method (ridge, lasso, OLS). By definition, the OLS race-specific slopes and fitted values are invariant to the constraints (ABCs or RGE), and `Over` cannot be computed for OLS.
